# Nutritional and Bioactive Characterization of *Amaranthaceae* Seeds From Peru, Slovakia, and Poland: A Comparative Study

**DOI:** 10.1002/fsn3.70901

**Published:** 2025-09-10

**Authors:** Emmanuel Duah Osei, Alfred Elikem Kwami Afedzi, Anthony Amotoe‐Bondzie, Eva Ivanišová, Christian R. Encina‐Zelada, Sylwester Czaplicki, Iveta Čičová, Ivona Jančo, Branislav Gálik, Newlove Akowuah Afoakwah

**Affiliations:** ^1^ School of Food Science and Environmental Health Technological University Dublin Dublin Ireland; ^2^ Sustainability and Health Research Hub Technological University Dublin Dublin Ireland; ^3^ Department of Biotechnology, Fermentation Technology Research Center, Faculty of Agro‐Industry Kasetsart University Bangkok Thailand; ^4^ Institute of Food Sciences, Faculty of Biotechnology and Food Sciences Slovak University of Agriculture in Nitra Nitra Slovakia; ^5^ Food Incubator, AgroBioTech Research Centre Slovak University of Agriculture Nitra Slovakia; ^6^ Department of Food Technology, Faculty of Food Industries Universidad Nacional Agraria La Molina Lima Peru; ^7^ Instituto de Investigación de Bioquímica y Biología Molecular (IIBBM) Universidad Nacional Agraria La Molina Lima Peru; ^8^ Plant Food Chemistry and Processing, Faculty of Food Sciences University of Warmia and Mazury in Olsztyn Olsztyn Poland; ^9^ National Agricultural and Food Centre Research Institute of Plant Production Piešťany Slovakia; ^10^ AgroBioTech Research Centrum Slovak University of Agriculture in Nitra Nitra Slovakia; ^11^ Institute of Nutrition and Genomics, Faculty of Agrobiology and Food Resources Slovak University of Agriculture in Nitra Nitra Slovak Republic; ^12^ Department of Food Science and Technology, Faculty of Agriculture, Food and Consumer Sciences, Nyankpala Campus University for Development Studies Tamale Ghana

**Keywords:** *Amaranthaceae*, amino acid profile, antioxidant activity, mineral composition, phytosterols and squalene content, tocopherols, total flavonoid and polyphenol content

## Abstract

*Amaranthus* sp. represents a promising pseudocereal due to its resilience to adverse environmental conditions and extensive nutritional benefits. This study evaluated amaranth samples from Peru, Slovakia, and Poland to compare their proximate composition, mineral content, amino acid profiles, antioxidant potential, and specific lipid fractions (phytosterols, squalene, and tocopherols). Results revealed significant regional variability: Polish cultivars generally exhibited the highest (*p* < 0.05) protein and squalene concentrations, while certain Peruvian kiwicha varieties excelled (*p* < 0.05) in total phenolic compounds and antioxidant capacity. Mineral analyses confirmed diverse calcium, magnesium, and iron levels, underscoring environmental and genetic influences. Amino acid profiling highlighted consistently high (*p* < 0.05) lysine and leucine, particularly in high‐protein samples, reinforcing amaranth's potential as a complementary protein source. Lipid analysis revealed substantial (*p* < 0.05) phytosterols (dominated by *β*‐sitosterol) and varying tocopherol isomers, with certain cultivars displaying notably high *δ*‐tocopherol. Correlation and principal component analyses further demonstrated trait clustering (e.g., protein, squalene, and essential amino acids), suggesting targeted breeding opportunities to optimize nutritional density and bioactive composition. These findings reaffirm amaranth's application in functional food development, particularly in gluten‐free applications, and emphasize its role as a strategic crop in addressing nutritional deficiencies and promoting dietary diversity.

## Graphical abstract

*Amaranthus* sp. seeds from Peru, Slovakia, and Poland showed significant variability in nutrients and bioactive compounds. Polish cultivars had the highest protein and squalene levels, while Peruvian kiwicha excelled in polyphenols and antioxidants. Slovak and Polish samples contained abundant phytosterols and distinct tocopherol isomers, reinforcing functional food potential. Trait clustering (protein, squalene, amino acids) suggests opportunities for targeted breeding. Findings support amaranth's role in gluten‐free, high‐protein formulations to enhance dietary diversity and nutrition.

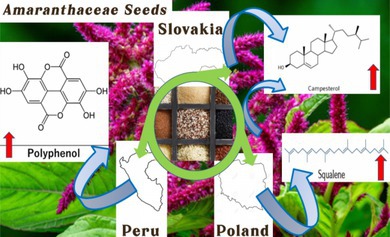

## 1 Introduction

Global climate change, rising global hunger, and evolving crop patterns in Europe and beyond have accelerated the search for nutritionally dense and health‐promoting crops. Pseudocereals, a diverse group of non‐grass plants used as staple foods worldwide, particularly in low‐income regions, have attracted renewed attention (Joshi et al. 2019). Among prominent pseudocereals such as buckwheat, sorghum, millet, chia, and quinoa, *Amaranthus* stands out for its robust environmental adaptability, including tolerance to drought and high temperatures (8°C–35°C) (Mukuwapasi et al. 2024; Netshimbupfe et al. 2023). This makes it an attractive candidate for cultivation in regions susceptible to environmental stress. Historically, amaranth has an ancient heritage in Mesoamerica, where it was a fundamental Aztec food crop known as Huautli or Xtes. Its modern resurgence began in the mid‐1970s, spurred by evidence of its superior nutritional attributes compared to conventional cereals (Soriano‐García and Aguirre‐Díaz 2019). This reveals its relevance in global food systems, particularly as consumer demand shifts toward naturally gluten‐free and high‐protein grains.

Amaranth's significance lies in its high‐quality protein, typically ranging from 13% to 19%, enriched with essential amino acids like lysine, which is often limited in cereal grains (Cornejo et al. 2019; Venskutonis and Kraujalis 2013). This gluten‐free pseudocereal also harbors substantial nutraceutical constituents, including unsaturated oils, squalene, dietary fiber, tocopherols, tocotrienols, phenolic compounds, and flavonoids. Furthermore, it exhibits remarkable adaptability to adverse environments, tolerating drought and heat while maintaining robust yields, traits that align well with the quest for climate‐resilient and nutritionally rich food sources (Alemayehu et al. 2015; Joshi et al. 2018; Rastogi and Shukla 2013).

Recent studies have drawn attention to amaranth's broad chemical composition, wide range biological activities, and potential for health promotion and pharmacological applications. Amaranth is recognized as a valuable functional food with the potential to enhance overall health and reduce the risk of metabolic disorders. Its notable therapeutic properties have supported traditional uses in managing conditions such as diarrhea, heavy menstrual bleeding, and intestinal bleeding (Caeiro et al. 2022).

Notably, different *Amaranthus* species show pronounced variation in amino acid composition, fatty acid profiles, mineral content, and other physicochemical characteristics (Gresta et al. 2020; Siwatch et al. 2019; Venskutonis and Kraujalis 2013). Considering the versatile applications of amaranth, from functional food formulations to gluten‐free products, further comparative analyses of cultivars from different regions can help identify genotypes with optimal nutritional and health‐promoting profiles.

Hence, this study aimed to evaluate and compare the proximate composition, mineral content, amino acid profile, antioxidant capacity, and select lipid components (phytosterols, squalene, and tocopherols) of *Amaranthu*s sp. originating from Peru, Slovakia, and Poland. These offer insights into how geographical and genetic factors shape amaranth's nutritional and functional properties and its role in addressing nutritional gaps and fostering global food security.

## 2 Materials and Methods

### 2.1 Plant Material

*Amaranthus* sp. seeds were obtained from three countries: Peru, Slovakia, and Poland, encompassing a total of 10 samples.

### 2.2 Peruvian Amaranth Varieties

Five Peruvian kiwicha ( *Amaranthus caudatus* L.) samples were collected: “Kiwicha ( *Amaranthus caudatus* L.)” has been cultivated for generations across various Andean regions of Peru: Apurímac, Cusco, Cajamarca, Huancayo, or Ayacucho. The crop thrives in marginal soils and under irregular rainfall conditions due to its natural resilience and well‐established traditional cultivation practices. Annual rainfall ranges from 500 to 900 mm, concentrated between November and March. It grows best in well‐drained loamy to clay‐rich soils, with organic matter typically maintained between 2% and 4%. Soil is slightly acidic to neutral (pH 6.0–7.5), with nitrogen present in modest amounts but improved through intercropping with legumes. In contrast, potassium and micronutrients like calcium, magnesium, and zinc are generally adequate due to natural soil weathering.

Peruvian varieties used were *Kiwicha red and white*: Sourced from Apurimac and Cusco (3399 m.a.s.l.; climate is temperate to cold, with wet summers and dry winters, soils are predominantly volcanic and alluvial). The sample was a mixture of red kiwicha and white kiwicha (Oscar Blanco variety), with diameters of 1.0–1.5 mm.

- *Kiwicha black and white*: Sourced from Cajamarca (2750 m.a.s.l.; subtropical highland climate with mild temperatures, volcanic soil) and Apurimac (3399 m.a.s.l.; climate is temperate to cold, with wet summers and dry winters, soils are predominantly volcanic and alluvial). The sample was a mixture of black kiwicha and white kiwicha (Oscar Blanco variety), with diameters of 1.0–1.5 mm.
- *Kiwicha Centenario variety*: Sourced from Huancayo (3259 m.a.s.l.; subtropical highland climate characterized by mild temperatures, soil of volcanic origin). This sample comprised white kiwicha Centenario seeds, with diameters of 1.0–1.5 mm.
- *Kiwicha local market 1*: Sourced from Cusco (3399 m.a.s.l.; climate is temperate to cold, with wet summers and dry winters, soils are predominantly volcanic and alluvial). The sample was the INIA 430—Imperial variety, with diameters of 1.0–1.5 mm; and
- Kiwicha local market 2: Sourced from Ayacucho (2761 m.a.s.l.; highland climate with dry, temperate conditions, volcanic and loamy soil). The sample was the INIA 413—Morocho variety, with diameters of 1.0–1.5 mm.

### 2.3 Slovak and Polish Amaranth Varieties

Amaranthus (Amaranthus L.) was sourced from Slovakia and Poland, which had light to medium, sandy loam, sandy loam, or light loam soils that do not tend to compact and are suited to a wide range of soil reactions from 5.5 to 7.3 pH. It requires much less water for growth than other crops (42%–45% of the needs of wheat, or 51%–62% of the needs of corn). For rapid germination and emergence, air temperatures above 15°C and soils warmed to 12°C are necessary. Plants are moderately resistant to cold; long‐term low temperatures below 4°C are critical for young plants. Minimum temperatures for the growth of emerged plants are 10°C–15°C. Fertilizer application to meet nutrient requirements averages a dose of N 30–90, P 20–30, K 25–60 kg ha^−1^ to suppress weeds, maintain soil moisture, and ensure good soil structure. Amaranth is sown when the soil temperature has reached 10°C–12°C. The soils are rich in minerals, including calcium, sodium, and potassium.

From Slovakia, three samples were obtained from the National Agricultural and Food Centre in Pieštany (162 m.a.s.l.; mild Mediterranean continental climate, the soils are predominantly brown soils and cambiums, which have good fertility and are suitable for agricultural production): Slovak varieties used were

- Variety Olpir ( *Amaranthus cruentus* L.)
- Variety Koniz ( *Amaranthus hypochondriacus* L.)
- Variety Zobor ( *Amaranthus hypochondriacus* × *Amaranthus hybridus* ).

*From Poland*, *Amaranthus cruentus* L. was used: (i) variety Rawa and (ii) variety Aztek; from Poland (locality Olsztyn 154 ma.s.l.; temperate climate with moderate temperatures, fertile soil consisting of loamy and sandy textures.), soil temperature 10°C–12°C. *Amaranthus cruentus* L. was used:

- Variety Rawa
- Variety Aztek.

Before analysis, all seeds were stored at room temperature and then ground using a Labconco 3100 grinder (Perten Instruments, Springfield, IL, USA) to achieve a uniform particle size.

### 2.4 Chemicals

All chemicals and reagents (analytical grade) were purchased from Sigma‐Aldrich (St. Louis, MO, USA) and CentralChem (Bratislava, Slovakia).

### 2.5 Sample Extraction for Antioxidant and Phenolic Analysis

For antioxidant assays (DPPH), total phenolic content, total flavonoid content, and total phenolic acids, 0.5 g of ground seed material was extracted with 20 mL of 80% ethanol for 2 h. The extract was centrifuged at 4000 × g for 10 min (Rotofix 32 A, Hettich, Germany). The supernatant was collected and used for subsequent analyses. All extractions were performed in triplicate.

### 2.6 Proximate Composition Analyses

#### 2.6.1 Dry Matter, Ash, Crude Protein, and Starch

Dry matter, ash, starch, and crude protein contents were determined according to the AACC (1996) Method 08–01. Nitrogen was measured by the semi‐micro Kjeldahl method, and crude protein content was calculated using a nitrogen conversion factor of 6.25.

#### 2.6.2 Fat Content

Fat content was determined using an Ancom XT15 fat extractor (Ancom, USA) following the manufacturer's instructions. Briefly, 1.5 g of the sample (*W*_1_) was placed in a filter bag (XT4, Ancom, USA) and dried at 105°C for 3 h to remove moisture. The bag was cooled in a desiccator and weighed (*W*_2_), then extracted with petroleum ether for 60 min at 90°C. After extraction, the sample was dried at 105°C for 30 min, cooled in a desiccator, and weighed again (*W*_3_). Fat content was calculated using the formula: Fat content (%) = [(*W*_2_ − *W*_3_)/*W*_1_] × 100.

### 2.7 Amino Acid Composition

The amino acid composition was analyzed on an Ingos (Prague, Czech Republic) amino acid analyzer using ion‐exchange chromatography with a strong cation‐exchange resin and a sodium‐citrate buffer system, followed by post‐column derivatization with ninhydrin and spectrophotometric detection. The instrument was calibrated with standard amino acid solutions. Tryptophan was excluded because it is degraded by acid hydrolysis, and asparagine and glutamine were measured as aspartic acid and glutamic acid, respectively, due to deamidation.

### 2.8 Mineral Composition

Mineral elements (Ca, Na, K, Mg, Al, Ba, Cu, Fe, Li, Mn, Sr., and Zn) were determined using inductively coupled plasma optical emission spectroscopy (ICP‐OES; Thermo iCAP Dual 6500, USA). Approximately 0.2 g of ground sample was subjected to microwave‐assisted digestion (Ethos One, Milestone, Italy) in Teflon vessels containing 8 mL of 65% HNO_3_. The digestion protocol lasted 1 h, with a temperature ramp not exceeding 200°C. Each digested sample was diluted to 50 mL with ultrapure water, and a blank (acid only) was run in parallel. Calibration was conducted using certified standard solutions (Merck): 10,000 ppm (Ca, Fe, K, Mg, and P) and 1000 ppm (Na, Al, Ba, Cu, Li, Mn, Sr., and Zn). A three‐point calibration curve was generated for each element. Internal standard correction (yttrium and ytterbium ions at 2–5 mg/L) was applied to minimize instrumental drift.

### 2.9 Bioactive Compounds Analysis

#### 2.9.1 DPPH Assay

The free radical scavenging activity was measured according to Yen and Chen (1995) with slight modifications. A solution of 0.012 g DPPH in 100 mL ethanol was prepared, and 4 mL of this solution was added to 1 mL of the ethanolic seed extract. After incubation, absorbance was read at 515 nm using a BioTek Microplate Reader (ELx800). Results are reported as mg of Trolox equivalent antioxidant capacity (TEAC) per gram of dry matter.

#### 2.9.2 Total Polyphenol Content

Total polyphenols were estimated using a modified Folin–Ciocalteu method (Singleton and Rossi 1965). Briefly, 0.1 mL of extract was mixed with 0.1 mL of Folin–Ciocalteu reagent and 1 mL of 20% sodium carbonate. After incubation, absorbance was measured at 700 nm (BioTek ELx800). Results are expressed in mg of gallic acid equivalent (GAE) per gram of dry matter.

#### 2.9.3 Total Flavonoid Content

Flavonoid content was determined following the method of Willett (2002) with minor modifications. A 0.5 mL aliquot of the extract was combined with 0.1 mL of 10% (w/v) aluminum chloride in ethanol, 0.1 mL of 1 M potassium acetate, and 4.3 mL of distilled water. After 30 min in the dark, absorbance was measured at 415 nm (Jenway 6405 UV–Vis, UK). Results are reported as mg quercetin equivalents (QE) per gram of dry matter.

#### 2.9.4 Total Phenolic Acid Content

Total phenolic acids were measured according to Jain et al. (2017). A 0.5 mL aliquot of extract was mixed with 0.5 mL of 0.5 M HCl, 0.5 mL of Arnova reagent (10% NaNO_2_ + 10% Na_2_MoO_4_), 0.5 mL of 1 M NaOH, and 0.5 mL of water. After mixing, the absorbance was read at 490 nm (Jenway 6405 UV–Vis, UK). Results are expressed as mg of caffeic acid equivalents (CAE) per gram of dry matter.

### 2.10 Phytosterols and Squalene Analysis

Phytosterols and squalene were quantified using gas chromatography–mass spectrometry (GC–MS) following a method adapted from Dąbrowski et al. (2018). First, 0.2 g of extracted oil was mixed with 0.2 mL of 5‐*α*‐cholestane (internal standard, 0.4 mg/g), saponified, and the unsaponifiable fraction was extracted. The fraction was derivatized with 100 μL of pyridine and 100 μL of BSTFA + 1% TMCS at 60°C for 60 min. Separation was performed on an Agilent 8890 GC system coupled with a 7000D MS detector using an HP‐5MS UI column (30 m × 250 μm × 0.25 μm). The carrier gas was helium at a flow rate of 0.9 mL/min. The injector temperature was 230°C. Oven conditions were: 70°C for 2 min, ramped to 230°C at 15°C/min, then ramped to 310°C at 3°C/min, and held for 10 min. The interface and ion source were at 240°C and 220°C, respectively, with an electron energy of 70 eV. Quantification was done via total ion current (TIC) in the m/z 50–600 range. Identification was based on retention times and the NIST20 library. The limits of quantification were 0.05 μg/g of oil, while the linearity of the calibration curves was confirmed in the range of 2.5–100 μg/mL with *R* ^2^ ≥ 0.98452.

### 2.11 Tocopherols Analysis

Tocopherols (*α‐*, *β‐*, *γ*‐, and *δ*‐) were measured by high‐performance liquid chromatography (HPLC; Agilent 1200 Series, USA) with a fluorescence detector, according to Czaplicki et al. (2016). The oil–in–n‐hexane solution (1%, m/v) was injected into the chromatographic system. Separation was performed on a Reprospher Si 100 column (200 mm × 3 mm, 3 μm; Dr. Maisch‐GmbH, Germany), using 0.7% isopropanol in n‐hexane at 1.0 mL/min. The fluorescence detector was set to *λ*_ex = 296 nm and *λ*_em = 330 nm. External standards of *α‐*, *β‐*, *γ*‐, and *δ*‐tocopherols (Merck, Darmstadt, Germany) were used for peak identification and quantification. Calibration curves had *R* ^2^ ≥ 0.9967. Repeatability was expressed as a coefficient of variation (< 3.68%). The limits of quantification were, depending on tocopherol, 0.2–0.45 μg/g of oil, while the linearity of the calibration curves was confirmed in the range of 0.02–16 μg/mL with *R* ^2^ ≥ 0.99925.

### 2.12 Statistical Analysis

All measurements were carried out in triplicate. Data are presented as mean ± standard deviation. Statistical analysis was performed using RStudio (v. 2023.09.1, Boston, MA, USA) software through the ‘*aov*’, ‘*lm*’, ‘*shapiro.test*’, ‘*bartlett. test*’, ‘*dwtest*’, ‘*HSD.test*’, or ‘*kruskal.test*’ functions. Prior to conducting inferential statistical tests, all datasets were evaluated for normality (Shapiro–Wilk test), homogeneity of variances (Bartlett's test), and independence of residuals (Durbin–Watson test). When assumptions of parametric analysis were not encountered, Box‐Cox transformations (*λ* ranging from −2.0 to +2.0) were applied to stabilize variance or approximate normal distribution of errors. Parametric tests (e.g., one‐way ANOVA followed by Tukey's HSD *post hoc* test) were used only when transformed data satisfied these assumptions. In cases where transformation did not achieve normality or homoscedasticity, non‐parametric alternatives (e.g., Kruskal–Wallis test with Dunn's post hoc test) were employed. All statistical analyses were conducted with a significance threshold of *p* < 0.05 and with a significance level of 5% (*α* = 0.05). Principal component analysis (PCA) was also conducted to visualize relationships among variables and sample groupings using ‘*FactoMineR*’, ‘*factoextra*’ and ‘*corrplot’* libraries.

## 3 Results and Discussion

### 3.1 Proximate Composition and Bioactive Compounds Analysis of *Amaranthus* Species

#### 3.1.1 Proximate Composition

Table 1 presents the crude protein, starch, fat, ash, and dry matter for the amaranth samples. Crude protein content ranged from 12.0 g/100 g (Kiwicha local market 2) to 18.3 g/100 g (Rawa) with significant (*p* < 0.05) differences. Polish varieties (Rawa and Aztek) generally exhibited higher protein content, whereas Peruvian samples varied more widely. The observed protein range aligns with earlier studies reporting 13%–19% protein in amaranth grains, reinforcing its reputation as a good plant‐based protein source relative to common cereals (Andini et al. 2013; Preetham Kumar et al. 2016). Particularly, the protein content of the Peruvian samples aligns with the content obtained by Imeri et al. (1987). Notably, protein content of *A. caudatus from Peru was reported to have increased* from 12.35% to 14.50% with the application of NPK fertilizers but had no effect on protein quality of other varieties (Imeri et al. 1987). Organic manure from kola pod husk, when applied in combination with NPK at a 75:25 ratio, significantly increased crude protein content by 19.8% and 14.9% in *Amaranthus cruentus* grown on two different soil types in Lagos, Nigeria (Makinde Esther et al. 2010). Considering these reports, it may be inferred that the differences in protein content of the amaranth varieties in this study may be attributed to genetic factors, environmental factors including altitudes, soil nutrients, and agronomic practices used such as fertilizer applications during cultivation (Assad et al. 2017; Imeri et al. 1987; Makinde Esther et al. 2010; Reyes‐Rosales et al. 2023). Specifically, the higher protein content observed in the Slovak and Polish varieties may be attributed to agronomic practices, such as fertilizer application, which increase nitrogen accumulation in the crop. Under the EC nutrition and health claim, the amaranth samples in the study can be categorized as a protein source with at least 12% of the energy value provided by amaranth samples (European Commission 2006). For food applications, amaranth can be used to enhance the protein content and provide essential amino acids to cereal‐based foods that are typically low in both protein and amino acids (Vega‐Gálvez et al. 2010). Phenolic compounds such as betacyanins, caffeoylisocitric acid, and chlorogenic acid, and antinutrients like phytic acids and tannins influence digestion and absorption processes by binding digestive enzymes and forming complexes with proteins that hinder the digestibility and bioavailability of the available protein (Rivero Meza et al. 2023). Processing such as grain dehulling reduces tannins, while extrusion has been shown to improve the digestibility of proteins in in vitro studies after the extrusion process in “Centenario” and “Oscar Blanco” varieties of Andean native grain, kiwicha (Repo‐Carrasco‐Valencia et al. 2009).

**TABLE 1:** Proximate analysis (crude protein, starch, fat, ash, dry matter) of different A*maranthaceae* samples.

Sample	Crude protein (g/100 g)	Starch (g/100 g)	Fat (g/100 g)	Ash (g/100 g)	Dry matter (g/100 g)
Kiwicha black and white	13.6 ± 0.06^ef^	58.7 ± 0.03^cd^	3.85 ± 0.14^abc^	2.32 ± 0.05^bcd^	89.9 ± 0.04^a^
Kiwicha red and white	13.8 ± 0.01^de^	57.4 ± 0.55^e^	4.18 ± 0.02^abc^	1.99 ± 0.06^d^	89.3 ± 0.29^ab^
Kiwicha local market 1	12.7 ± 0.33^fg^	59.2 ± 0.19^c^	3.40 ± 0.10^c^	2.07 ± 0.08^d^	88.6 ± 0.56^b^
Kiwicha local market 2	12.0 ± 0.32^g^	62.1 ± 0.20^ab^	2.23 ± 0.50^d^	2.22 ± 0.10^cd^	89.5 ± 0.33^ab^
Kiwicha Centenario	15.5 ± 1.39^c^	61.5 ± 0.53^b^	3.55 ± 0.46^bc^	2.69 ± 0.19^a^	90.3 ± 0.42^a^
Pribina	17.3 ± 0.23^b^	63.0 ± 0.17^a^	3.94 ± 0.39^abc^	2.24 ± 0.04^cd^	90.5 ± 0.32^a^
Olpir	15.3 ± 1.18^cd^	57.4 ± 0.06^e^	4.48 ± 0.11^ab^	2.56 ± 0.09^abc^	90.0 ± 0.28^a^
Koniz	16.4 ± 0.01^c^	58.0 ± 0.03^de^	3.68 ± 0.06^bc^	2.61 ± 0.01^ab^	90.2 ± 0.10^a^
Rawa	18.3 ± 0.32^a^	49.5 ± 0.16^g^	4.83 ± 0.06^a^	2.05 ± 0.05^d^	90.0 ± 0.39^a^
Aztek	17.9 ± 0.01^ab^	53.7 ± 0.05^f^	4.77 ± 0.15^a^	2.12 ± 0.01^d^	89.6 ± 0.08^ab^
Shapiro–Wilk	0.025	0.2827	0.3987	0.9844	0.3666
Bartlett Test	0.0054	0.2448	0.2924	0.6276	0.7656
Durbin–Watson	0.463	0.9861	0.6862	0.2319	0.9974
Box‐Cox (λ)	0.9091	—	—	—	—
Tukey's HSD test	No	Yes	Yes	Yes	Yes
Kruskal–Wallis test	Yes	No	No	No	No

*Note:* Data are expressed as mean ± standard deviation (*n* = 3). *p*‐values are reported for the Shapiro–Wilk (normality), Bartlett (homoscedasticity), and Durbin–Watson (errors' independence) tests. Box–Cox lambda “*λ*” values (−2.0 to +2.0) indicate data transformation for normality or homoscedasticity. Different lowercase letters (^a–i^) denote statistically significant differences among amaranth samples using Tukey's HSD (parametric) or Dunn's test following Kruskal–Wallis (non‐parametric) analysis (*p* < 0.05), depending on assumption acquiescence.

The starch content differed significantly (*p* < 0.05) from 49.5 g/100 g (Rawa) to 63.0 g/100 g (Pribina). Starch values reported in this study were lower than 65%–75% starch reported to be generally contained in amaranth seeds based on processing methods and agronomic practices applied (Bojórquez‐Velázquez et al. 2018; Venskutonis and Kraujalis 2013). This discrepancy may be attributed to varietal differences or the presence of non‐endosperm components in the whole grain samples. Notably, González et al. (2007) demonstrated that higher starch concentrations, up to 79%, can be obtained through differential milling, which isolates the endosperm or removes the germ and bran fractions. Such high‐starch fractions are not representative of whole grain compositions, which may likely explain the relatively lower values observed in this study. Like proteins in amaranths, starch digestibility and bioavailability have been improved in an in vitro study through extrusion processing in “Centenario” and “Oscar Blanco” varieties of *A. caudatus* (Repo‐Carrasco‐Valencia et al. 2009). Variations in starch levels can profoundly affect the functional properties of grains, influencing texture and potential uses in gluten‐free or high‐energy formulations (Horstmann et al. 2017; Liu et al. 2019).

Fat content ranged between 2.23 and 4.83 g/100 g (Rawa), lower than the 5.80%–6.9% (Bojórquez‐Velázquez et al. 2018; Singh and Punia 2020) range found in most amaranth species. Differences in varieties studied and geographical location may account for these reported differences. Although relatively low, amaranth lipids are noted for their favorable fatty acid profile and presence of nutraceutical compounds like squalene, which is known for its hepatoprotective effects (Mondor et al. 2020; Szwejkowska and Bielski 2012). Ash levels varied slightly (1.99–2.69 g/100 g), with Kiwicha Centenario exhibiting the highest ash content, suggesting possible enrichment in essential micronutrients. The ash content was lower than 2.90%–3.70% reported by Bojórquez‐Velázquez et al. (2018). Dry matter content remained consistent (88.6–90.5 g/100 g), which may be indicative of similar post‐harvest processing and storage conditions.

While the proximate composition of amaranth supports its nutritional value, variability arising from genotype, environmental conditions, and processing methods must be acknowledged. Furthermore, antinutritional factors and limited bioavailability can constrain nutrient uptake, particularly in unprocessed forms. These challenges highlight the need for standardization, processing optimization, and further bioefficacy studies to fully realize the nutritional potential of amaranth.

#### 3.1.2 Antioxidant Capacity

**FIGURE 1:**
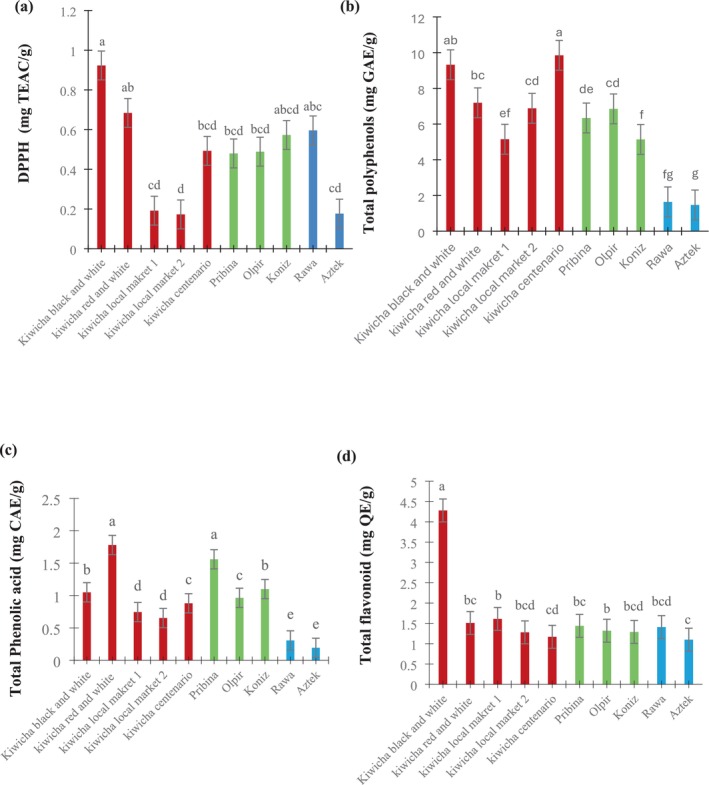
Antioxidant activity of different A*maranthaceae* samples: (a) Total phenolic acid (mg CAE/g), (b) total flavonoid (mg QE/g), (c) DPPH (mg TEAC/g), and (d) total polyphenols (mg GAE/g).

Significant differences (*p* < 0.05) were observed among the three antioxidant measures: total phenolic acids (0.193–1.78 mg CAE/g), total flavonoids (1.10–4.28 mg QE/g), and total polyphenols (1.47–9.85 mg GAE/g). As shown in Figure 1, Kiwicha red and white contained significantly higher phenolic acid levels (1.78 mg CAE/g) compared to Aztek (0.193 mg CAE/g), while Kiwicha black and white exhibited the greatest total flavonoid content (4.28 mg QE/g, *p* < 0.05). Total polyphenols peaked in Kiwicha Centenario at 9.85 mg GAE/g. A moderate positive correlation (*r* = 0.736) between starch and total polyphenols (Table 5) suggests that some high‐starch cultivars may also accumulate more phenolics, potentially as protective responses to environmental stress (Pandey 2018).

In our study, the total polyphenol values are generally higher than those reported for the Durga amaranth cultivar from India (Thakur et al. 2021). However, it is important to recognize that antioxidant measurements can vary widely due to differences in extraction solvents, assay methods, growing conditions, and sample maturity. For instance, some studies have reported lower phenolic contents in amaranth seeds grown under drought or nutrient‐deficient conditions (Stănilă et al. 2019), while others found higher values linked to specific genotypes or post‐harvest handling (Soriano‐García and Aguirre‐Díaz 2019) These sources of variability underscore the complexity of antioxidant profiling and limit broad generalizations across growing conditions and regions.

DPPH radical scavenging activity ranged from 0.173 mg TEAC/g (Kiwicha local market 2) to 0.923 mg TEAC/g (Kiwicha black and white). This variation commonly reflects differences in phenolic content, especially flavonoids, consistent with previous findings (Zhang et al. 2018). The robust DPPH activity in Kiwicha black and white may be attributed to its elevated flavonoid levels, highlighting the role of pigment‐related compounds in amaranth's antioxidant potential. DPPH values obtained for Polish Rawa and Aztek varieties were higher than reported by Paśko et al. (2009), possibly due to differences in extraction methods, solvent polarity, or growing conditions that may have influenced the antioxidant activity. While the antioxidant potential of amaranth is evident from the high levels of polyphenols and flavonoids observed in certain cultivars, the discussion predominantly emphasizes these positive attributes. However, it is important to acknowledge limitations that may affect both the interpretation and application of the findings. DPPH assays primarily measure radical scavenging under specific conditions and may not fully represent antioxidant behavior in biological systems due to factors such as bioavailability, metabolic transformation, and tissue‐specific effects, indicating a limitation of this method.

Phenolic antioxidants are known to mitigate oxidative stress and inflammation, potentially preventing chronic diseases such as cancer, cardiovascular disease, and diabetes, while also exhibiting antimicrobial and antiallergenic effects (Shahidi and Zhong 2015). Despite these promising bioactivities, further research using in vivo models and clinical trials is necessary to substantiate these health benefits in humans.

### 3.2 Mineral Composition

#### 3.2.1 Major Minerals (Ca, K, Mg, and Na)

In general, the mineral profile (Table 2) reveal notable cultivar‐ and region‐dependent variations, consistent with prior findings on pseudocereals (Gupta and Mishra 2024; Rodríguez et al. 2020). For instance, among the macro‐elements, Ca content ranged from 1413 mg/kg (Kiwicha local market 2) to 2825 mg/kg (Aztek). The higher Ca concentration in the Polish variety (Aztek) might result from its breeding lineage or soil characteristics in cultivation zones (Das and Das 2016). However, the bioavailability of calcium in plant‐based sources like amaranth is often limited due to the presence of antinutritional factors such as phytates and oxalates, which can bind calcium and reduce its absorption in the human gastrointestinal tract (Gibson et al. 2010). Nevertheless, some studies suggest that certain amaranth varieties have lower phytate‐to‐mineral molar ratios, potentially improving Ca bioavailability compared to other cereals (Kiewlicz and Rybicka 2020).

**TABLE 2:** Mineral composition (Ca, Na, K, Mg, Al, Ba, Cu, Fe, Li, Mn, Sr., and Zn), of different *Amaranthaceae* samples.

Sample	Ca	Na	K	Mg	Al	Ba	Cu	Fe	Li	Mn	Sr	Zn
Kiwicha black and white	2415 ± 7.03^d^	241 ± 1.00^d^	2935 ± 15.9^i^	208 ± 0.50^f^	251 ± 1.80^a^	12.6 ± 0.05^a^	4.06 ± 0.02^g^	132 ± 0.27^a^	0.100 ± 0.0017^a^	47.5 ± 0.09^b^	5.61 ± 0.03^h^	14.9 ± 0.689^e^
Kiwicha red and white	1417 ± 1.36^i^	175 ± 0.90^h^	2719 ± 8.02^j^	166 ± 0.35^j^	89.3 ± 0.94^c^	6.93 ± 0.02^c^	5.00 ± 0.03^f^	79.0 ± 0.42^d^	0.028 ± 0.0025^c^	25.4 ± 0.044^c^	7.40 ± 0.02^d^	25.9 ± 0.19^c^
Kiwicha local market 1	1452 ± 4.32^h^	216 ± 0.21^f^	3342 ± 2.04^f^	191 ± 0.27^h^	38.9 ± 0.42^e^	6.12 ± 0.01^d^	5.14 ± 0.02^e^	70.7 ± 0.29^e^	0.016 ± 0.0005^d^	20.5 ± 0.068^e^	9.80 ± 0.02^a^	18.9 ± 0.87^d^
Kiwicha local market 2	1413 ± 3.27^i^	226 ± 0.85^e^	3155 ± 8.17^g^	167 ± 0.19^i^	17.5 ± 0.26^g^	4.57 ± 0.02^f^	4.10 ± 0.04^g^	56.3 ± 0.20^h^	0.01098 ± 0.0009^ef^	16.0 ± 0.03^h^	7.30 ± 0.02^e^	20.5 ± 0.56^d^
Kiwicha Centenario	1573 ± 2.66^g^	254 ± 0.35^c^	3656 ± 6.97^d^	215 ± 0.14^e^	25.2 ± 0.50^f^	3.73 ± 0.01^g^	3.28 ± 0.04^i^	62.2 ± 0.23^g^	0.029 ± 0.001^c^	20.2 ± 0.03	8.26 ± 0.012^b^	20.9 ± 0.41^d^
Pribina	2081 ± 7.65^f^	370 ± 1.46^a^	3021 ± 4.22^h^	197 ± 0.19^g^	154 ± 2.09^b^	11.4 ± 0.01^b^	6.39 ± 0.02^b^	133 ± 0.47^a^	0.0858 ± 0.0019^b^	24.2 ± 0.032^d^	7.54 ± 0.007^c^	40.2 ± 0.49^a^
Olpir	2545 ± 4.23^c^	240 ± 0.26^d^	3376 ± 1.98^e^	248 ± 0.25^c^	19.95 ± 0.29^g^	4.95 ± 0.006^e^	6.66 ± 0.03^a^	83.1 ± 0.51^c^	0.0135 ± 0.0027^de^	19.5 ± 0.03^f^	5.91 ± 0.0054^g^	29.4 ± 1.11^b^
Koniz	2695 ± 3.94^b^	261 ± 0.71^b^	3861 ± 8.89^c^	266 ± 0.45^b^	10.7 ± 0.15^h^	3.60 ± 0.006^h^	5.99 ± 0.04^c^	52.5 ± 0.15^i^	0.0134 ± 0.0003^de^	17.1 ± 0.027^g^	6.42 ± 0.199^f^	24.4 ± 1.92^c^
Rawa	2236 ± 3.14^e^	206 ± 0.68^g^	3957 ± 10.2^b^	229 ± 0.47^d^	52.7 ± 1.08^d^	2.11 ± 0.013^i^	3.62 ± 0.015^h^	90.9 ± 0.13^b^	0.0248 ± 0.002^c^	14.1 ± 0.06^i^	3.88 ± 0.011^j^	23.7 ± 0.20^c^
Aztek	2825 ± 7.55^a^	254 ± 1.12^c^	4618 ± 11.5^a^	338 ± 0.62^a^	13.1 ± 0.77^h^	0.033 ± 0.008^j^	5.30 ± 0.01^d^	65.4 ± 0.08^f^	0.0066 ± 0.0006^f^	90.0 ± 0.26^a^	4.32 ± 0.0170^i^	31.7 ± 1.01^b^
Shapiro–Wilk	0.8977	0.9558	0.8902	0.7863	0.07412	0.03589	0.2879	0.9459	0.0452	0.00034	< 0.000001	0.3964
Bartlett Test	0.618	0.4003	0.3217	0.6773	0.03003	0.05521	0.8493	0.4563	0.1986	0.0116	< 0.000001	0.1274
Durbin–Watson	0.5154	0.9284	0.7111	0.9896	0.6193	0.9517	0.9562	0.7534	0.558	0.9867	0.3046	0.7634
Box‐Cox (λ)	—	—	—	—	0.6667	0.6667	—	—	0.6667	0.6667	0.6667	—
Tukey's HSD test	Yes	Yes	Yes	Yes	Yes	Yes	Yes	Yes	Yes	Yes	No	Yes
Kruskal–Wallis test	No	No	No	No	No	No	No	No	No	No	Yes	No

*Note:* Data are expressed as mean ± standard deviation (*n* = 3). *p*‐values are reported for the Shapiro–Wilk (normality), Bartlett (homoscedasticity), and Durbin–Watson (errors' independence) tests. Box–Cox lambda “*λ*” values (−2.00 to +2.00) indicate data transformation for normality or homoscedasticity. Different lowercase letters (^a–i^) denote statistically significant differences among amaranth samples using Tukey's HSD (parametric) or Dunn's test following Kruskal–Wallis (non‐parametric) analysis (*p* < 0.05), depending on assumption acquiescence.

K, an essential electrolyte for maintaining fluid balance and blood pressure regulation, was highest in Aztek (4618 mg/kg) and lowest in Kiwicha red and white (2719 mg/kg). As potassium in plant matrices is largely present in soluble forms, its bioavailability is typically high, often exceeding 80%–90% (FAO 2001) making amaranth a promising dietary source.

Mg varied markedly across samples (166–338 mg/kg), with Aztek again showing the highest concentration (338 mg/kg). A positive correlation between Mg and crude protein (*r* = 0.664, Table 5) suggests that cultivars with improved protein content may also contain higher Mg levels, possibly reflecting the role of Mg in enzymatic pathways. Although magnesium absorption can be affected by dietary components like fiber and phytate, its overall bioavailability remains moderate, with estimated absorption rates between 30% and 50% depending on individual physiological status and meal composition (Rosanoff et al. 2012).

#### 3.2.2 Minor and Trace Elements (Fe, Zn, Mn, cu, li, Sr., Ba, and Al)

The relatively high Fe (133 mg/kg) in these cultivars (Pribina and Kiwicha black and white) is noteworthy, given that iron deficiency remains prevalent in many regions. The cultivars had higher iron content than the Durga amaranth cultivar studied by Thakur et al. (2021). The differences in Zn uptake, 14.9 mg/kg (Kiwicha black and white) to 40.2 mg/kg (Pribina) might be influenced by soil pH and organic matter, which can affect Zn bioavailability (Reshma and Meenal 2024). These wide ranges align with prior research on amaranth's capacity to accumulate essential trace elements (Biel et al. 2017; Byrnes et al. 2017; Reshma and Meenal 2024). Manganese (Mn) was highest in Aztek (90.0 mg/kg), which might reflect both its genetic background and environmental growing conditions. The concentrations of aluminum (Al) and barium (Ba) showed substantial variation across the ten amaranth (Kiwicha) samples (Table 2). Al content ranged from 10.7 mg/kg (Koniz) to 251 mg/kg (Kiwicha black and white), while Ba ranged from 0.033 mg/kg (Aztek) to 12.6 mg/kg (Kiwicha black and white). These differences likely reflect environmental influences such as soil mineral content, irrigation water, and anthropogenic contamination (e.g., fertilizer use or atmospheric deposition). Although Al and Ba are nonessential, their presence in foodstuffs may raise toxicological concerns. Aluminum, particularly in its soluble forms, has been linked to neurotoxicity, oxidative stress and has been studied for its possible association with Alzheimer's disease (Kawahara and Kato‐Negishi 2011). While typical dietary Al intake is considered low risk in healthy individuals, concentrations exceeding 1 mg/kg body weight/week may be problematic, especially in vulnerable populations (EFSA 2008).

Barium, though naturally present in soils, can affect cardiovascular function and renal health if ingested at high doses over time. The WHO guideline for barium in drinking water is 0.7 mg/L, and although food‐based limits are less defined, high Ba intake from cereals may warrant monitoring in areas with known soil enrichment (FAO 2001). The Kiwicha black and white sample contained the highest concentrations of both Al and Ba, while Aztek consistently showed the lowest levels—indicating cultivar‐dependent uptake patterns.

Mineral‐rich grains such as amaranth may contribute to addressing micronutrient deficiencies, particularly in regions with limited access to animal‐based products. Moreover, the moderate to high contents of Fe, Zn, and Mg strengthen amaranth's appeal as a functional food ingredient aimed at improving mineral intake. It must be noted that factors like varietal selection, soil management, and processing method can further modify mineral bioavailability. Bioavailability challenges in amaranth primarily stem from its high levels of antinutrients such as phytates, tannins, and oxalates, which can bind minerals like iron, zinc, and calcium, reducing their absorption. Additionally, amaranth contains saponins and trypsin inhibitors that may impair nutrient digestion (Sidorova et al. 2023). Minerals such as magnesium, zinc, and manganese are more readily absorbed; however, the bioavailability of calcium in amaranth may be limited by the presence of oxalates (Sidorova et al. 2023). To enhance nutrient absorption from amaranth, processing methods such as soaking, sprouting, or fermenting are recommended to reduce antinutrients (Thakur et al. 2021). Consuming amaranth with vitamin C‐rich foods can further improve iron absorption (Castro‐Alba et al. 2019).

### 3.3 Amino Acid Composition

#### 3.3.1 Essential Amino Acids

Leucine (Leu), isoleucine (Ile), valine (Val), lysine (Lys), methionine (Met; not directly measured here), threonine (Thr), phenylalanine (Phe), and histidine (His) are among the essential amino acids crucial for human nutrition. Amaranth seeds generally present a well‐balanced profile of essential amino acids, notably lysine and leucine, which consistently appear at higher concentrations than most cereal grains (Table 3). Lysine ranged from 6.05 g/100 g protein (Kiwicha black and white) to 8.12 g/100 g protein (Aztek), making the composition of amaranth advantageous for supplementing lysine‐deficient staples. Leucine strongly correlates with total protein (*r* = 0.858, Table 5), particularly in Polish (Rawa, Aztek) and some Slovak cultivars (Koniz, Olpir), which exhibit remarkable essential amino acid density and reinforce amaranth's potential in protein‐enriched food products (Paredes‐Escobar et al. 2023; Patil et al. 2024). Notably, some amaranth proteins exhibit antioxidant activity, particularly peptides rich in hydrophobic amino acids such as leucine (Leu), alanine (Ala), and phenylalanine (Phe). These bioactive peptides and proteins, in turn, can exhibit a wide range of physiological effects, including antioxidant, antihypertensive, hypocholesterolemic, and immunostimulant activities (Bachar et al. 2020). According to Jan et al. (2023), amaranth seeds have a balanced distribution and high bioavailability of amino acids, and cooking can increase the bioavailability of its antioxidants. This warrants further studies that will investigate the impact of cooking methods on the bioavailability of amaranth samples.

**TABLE 3:** Amino acid composition (mg/100 g) of different *Amaranthaceae* samples.

Sample	asp	thr	ser	glu	pro	gly	ala	val	ile	leu	tyr	phe	his	lys	arg
Kiwicha black and white	7.41 ± 0.00^g^	2.37 ± 0.00^i^	5.15 ± 0.00^i^	18.6 ± 0.00^i^	0.00^a^	8.69 ± 0.00^g^	4.19 ± 0.00^i^	3.93 ± 0.00^h^	3.19 ± 0.00^i^	6.21 ± 0.00^i^	3.62 ± 0.00^i^	5.73 ± 0.00^e^	2.78 ± 0.00^i^	6.15 ± 0.00^i^	9.53 ± 0.00^i^
Kiwicha red and white	6.94 ± 0.00^i^	2.81 ± 0.00^h^	5.26 ± 0.00^h^	21.3 ± 0.00^g^	0.00^a^	8.61 ± 0.00^h^	4.80 ± 0.00^e^	4.33 ± 0.00^e^	3.55 ± 0.00^f^	6.91 ± 0.00^f^	4.42 ± 0.00^g^	5.94 ± 0.00^c^	3.09 ± 0.00^f^	7.18 ± 0.00^g^	10.4 ± 0.00^g^
Kiwicha local market 1	7.82 ± 0.00^f^	4.85 ± 0.00^d^	7.51 ± 0.00^e^	22.2 ± 0.00^e^	0.00^a^	9.19 ± 0.00^e^	4.42 ± 0.00^g^	4.40 ± 0.00^d^	3.54 ± 0.00^g^	6.81 ± 0.00^g^	4.57 ± 0.00^e^	5.61 ± 0.00^f^	3.08 ± 0.00^g^	7.52 ± 0.00^e^	10.5 ± 0.00^f^
Kiwicha local market 2	7.18 ± 0.00^h^	4.39 ± 0.00^g^	6.19 ± 0.00^g^	20.8 ± 0.00^h^	0.00^a^	8.48 ± 0.00^i^	4.36 ± 0.00^h^	4.07 ± 0.00^g^	3.29 ± 0.00^h^	6.37 ± 0.00^h^	3.85 ± 0.00^h^	5.42 ± 0.00^g^	2.90 ± 0.00^h^	7.05 ± 0.00^h^	10.1 ± 0.00^h^
Kiwicha Centenario	7.96 ± 0.00^e^	4.94 ± 0.00^c^	8.05 ± 0.00^b^	23.8 ± 0.00^c^	0.00^a^	9.18 ± 0.00^f^	4.85 ± 0.00^d^	4.64 ± 0.00^c^	3.78 ± 0.00^d^	7.24 ± 0.00^d^	4.63 ± 0.00^d^	5.99 ± 0.00^b^	5.53 ± 0.00^a^	7.78 ± 0.00^d^	11.3 ± 0.00^e^
Pribina	7.96 ± 0.00^e^	4.94 ± 0.00^a^	8.05 ± 0.00^b^	23.8 ± 0.00^c^	0.00^a^	9.18 ± 0.00^f^	4.85 ± 0.00^d^	4.64 ± 0.00^c^	3.78 ± 0.00^d^	7.24 ± 0.00^d^	4.63 ± 0.00^d^	5.99 ± 0.00^b^	5.53 ± 0.00^a^	7.78 ± 0.00^d^	11.3 ± 0.00^e^
Olpir	8.67 ± 0.00^c^	5.36 ± 0.00^b^	7.46 ± 0.00^f^	22.0 ± 0.00^f^	0.00^a^	9.31 ± 0.00^d^	4.69 ± 0.00^f^	4.25 ± 0.00^f^	3.64 ± 0.00^e^	6.96 ± 0.00^e^	4.45 ± 0.00^f^	5.76 ± 0.00^d^	3.13 ± 0.00^e^	7.44 ± 0.00^f^	11.4 ± 0.00^d^
Koniz	8.53 ± 0.00^d^	5.49 ± 0.00^a^	8.54 ± 0.00^a^	24.7 ± 0.00^b^	0.00^a^	10.1 ± 0.00^b^	5.01 ± 0.00^c^	4.64 ± 0.00^b^	3.89 ± 0.00^c^	7.41 ± 0.00^c^	4.74 ± 0.00^c^	6.40 ± 0.00^a^	3.34 ± 0.00^d^	7.91 ± 0.00^b^	12.9 ± 0.00^a^
Rawa	11.1 ± 0.00^a^	4.70 ± 0.00^e^	7.60 ± 0.00^d^	24.8 ± 0.00^a^	0.00^a^	10.2 ± 0.00^a^	5.11 ± 0.00^b^	5.41 ± 0.00^a^	4.96 ± 0.00^a^	7.46 ± 0.00^b^	4.74 ± 0.00^b^	5.41 ± 0.00^h^	3.97 ± 0.00^c^	7.81 ± 0.00^c^	12.8 ± 0.00^b^
Aztek	10.9 ± 0.00^b^	4.44 ± 0.00^f^	7.63 ± 0.00^c^	22.3 ± 0.00^d^	0.00^a^	10.0 ± 0.00^c^	5.24 ± 0.00^a^	5.41 ± 0.00^a^	4.93 ± 0.00^b^	7.62 ± 0.00^a^	4.91 ± 0.00^a^	5.36 ± 0.00^i^	3.98 ± 0.00^b^	8.12 ± 0.00^a^	12.4 ± 0.00^c^
Shapiro–Wilk	< 0.00001	< 0.00001	< 0.00001	< 0.00001	—	< 0.00001	< 0.00001	< 0.00001	< 0.00001	< 0.00001	0.001417	< 0.00001	0.001867	< 0.00001	< 0.00001
Bartlett Test	< 0.00001	< 0.00001	< 0.00001	< 0.00001	—	< 0.00001	< 0.00001	< 0.00001	< 0.00001	< 0.00001	< 0.00001	< 0.00001	< 0.00001	< 0.00001	< 0.00001
Durbin–Watson	0.185	0.1569	0.4062	0.09624	—	0.08473	0.1143	0.58	0.2908	0.1528	0.03532	0.1502	0.05798	0.1256	0.1644
Box‐Cox (λ)	—	—	—	—	—	—	—	—	—	—	—	—	—	—	—
Tukey's HSD test	No	No	No	No	—	No	No	No	No	No	No	No	No	No	No
Kruskal–Wallis test	Yes	Yes	Yes	Yes	—	Yes	Yes	Yes	Yes	Yes	Yes	Yes	Yes	Yes	Yes

*Note:* Data are expressed as mean ± standard deviation (*n* = 2). *p*‐values are reported for the Shapiro–Wilk (normality), Bartlett (homoscedasticity), and Durbin–Watson (errors' independence) tests. Box–Cox lambda “*λ*” values (−2.00 to +2.00) indicate data transformation for normality or homoscedasticity. Different lowercase letters (^a–i^) denote statistically significant differences among amaranth samples using Tukey's HSD (parametric) or Dunn's test following Kruskal–Wallis (non‐parametric) analysis (*p* < 0.05), depending on assumption acquiescence.

#### 3.3.2 Non‐Essential Amino Acids

Among non‐essential amino acids (NEAAs), glutamic acid (Glu) consistently dominated, ranging from 18.6 g/100 g protein (Kiwicha black and white) to 24.8 g/100 g protein (Rawa). While these amino acids contribute to sensory attributes such as umami taste and functional properties like protein solubility and buffering capacity (Jinap and Hajeb 2010; Tan et al. 2023), the nutritional implications of these high levels are limited, given that NEAAs can be synthesized endogenously in healthy individuals. The undetection of proline (Pro) in several samples may reflect genotype‐specific expression or stress‐related suppression, yet this remains speculative due to the multiseason sampling. Given that proline accumulation is often stress‐induced, its variability warrants deeper investigation under defined agronomic conditions and cultivation seasons (Luyckx et al. 2021). Other non‐essential amino acids like alanine (Ala), glycine (Gly), and serine (Ser) showed moderate ranges. High lysine content, combined with favorable distributions of other essential amino acids, shows amaranth's capacity to complement lysine‐deficient staples and enhance overall protein quality. Even though the presence of these amino acids contributes to the overall protein matrix, their biological importance in the human diet is generally lower compared to essential amino acids (EAAs), and their health‐promoting roles have not been explored. Statistical analyses further revealed significant (*p* < 0.05) associations (Table 5) between protein, lysine, and certain minerals (e.g., magnesium), suggesting that high‐protein cultivars may simultaneously offer good mineral and amino acid (Luyckx et al. 2021).

The high amino acid content of the amaranth samples may equally show enhanced bioactivity and bioavailability. For example, peptides have been obtained from amaranth proteins showing strong antioxidant and anti‐inflammatory potential (Sandoval‐Sicairos et al. 2021). To maximize the bioactivity and bioavailability of amaranth proteins, extractions with protease enzymes may prove vital for releasing bioactive peptides and amino acids. In this context, *in silico* approaches can enhance efficiency and specificity by guiding enzyme selection and cleavage site prediction, ultimately improving peptide bioactivity and the bioavailability of amino acids (Osei et al. 2025).

### 3.4 Phytosterols and Squalene

Squalene content in the analyzed amaranth seeds ranged from 1392 mg/100 g in Kiwicha black and white to 4452 mg/100 g in Koniz and 4250 mg/100 g in Aztek, suggesting that certain Slovak (Koniz) and Polish (Aztek) cultivars may be richer sources of this bioactive compound (Table 4). Squalene concentrations observed for samples in this study were higher than reported for wild and cultivated amaranth seed varieties, including *A. cruentus* , *A. powellii* , *and A. hypochondriacus cv Opaca* (Bojórquez‐Velázquez et al. 2018). This variation may be attributed to genotypic differences among varieties, environmental factors such as soil quality and climate, or post‐harvest handling conditions. Additionally, differences in extraction methods and analytical sensitivity may contribute to the higher values observed in the present study. Squalene is recognized for its antioxidant activity and possible roles in lipid metabolism, hepatic protection, and anti‐inflammatory processes, supporting its use in functional food formulations (Antonio and Javier 2019; Baraniak and Kania‐Dobrowolska 2022; Zehravi et al. 2022). Processing of amaranth seeds may have some influence on the distribution and content of squalene. The processing stability of squalene in amaranth was demonstrated, with no loss during puffing (up to 290°C) and a maximum loss of 12% during roasting at 150°C for 20 min, indicating high thermal stability in oil‐rich fractions (Tikekar et al. 2008). That notwithstanding, squalene's biological effects and stability during processing or digestion remain active areas of investigation, and extrapolation to human health benefits requires caution without in vivo or clinical validation.

**TABLE 4:** Phytosterols (squalene, campesterol, stigmasterol, *β*‐sitosterol, and isofucosterol, in mg/100 g) and tocopherols (*α‐*, *β‐*, *γ‐*, and *δ‐*, in mg/100 g) composition of different *Amaranthaceae* samples.

Sample	Squalene	Campesterol	Stigmasterol	*β*‐sitosterol	Isofucosterol	*α*‐tocopherols	*β*‐tocopherols	*γ*‐tocopherols	*δ*‐tocopherols
Kiwicha black and white	1392 ± 10.8^g^	70 ± 2.97^de^	191 ± 13.0^de^	170.1 ± 15.3^c^	76.5 ± 3.82^c^	0.0 ± 0.00^f^	32.5 ± 2.09^b^	0.0 ± 0.00^c^	10.2 ± 0.64^g^
Kiwicha red and white	2035 ± 15.0^f^	54.7 ± 0.92^e^	157 ± 3.96^e^	109.5 ± 2.05^d^	62.1 ± 2.83^def^	0.0 ± 0.00^f^	48.7 ± 0.21^a^	0.0 ± 0.00^c^	1786 ± 19.2^a^
Kiwicha local market 1	2681 ± 31.3^e^	69.5 ± 6.58^de^	165 ± 5.44^e^	143.9 ± 9.69^cd^	72.2 ± 1.06^cd^	2.91 ± 0.22^e^	48.4 ± 1.65^a^	0.0 ± 0.00^c^	12.4 ± 0.26^e^
Kiwicha local market 2	3021 ± 102^de^	65.7 ± 3.32^de^	155 ± 8.13^e^	116.9 ± 9.26^d^	66.8 ± 1.98^cde^	3.49 ± 0.18^d^	45.9 ± 1.85^a^	0.0 ± 0.00^c^	11.7 ± 0.50^f^
Kiwicha Centenario	2855 ± 270^e^	157 ± 0.99^c^	311 ± 14.8^c^	258.1 ± 16.1^b^	109 ± 3.68^b^	3.44 ± 0.01^d^	49.3 ± 1.18^a^	0.0 ± 0.00^c^	970 ± 24.17^b^
Pribina	3693 ± 13.4^bc^	57.4 ± 2.90^e^	185 ± 12.5^de^	62.7 ± 2.33^e^	33.1 ± 1.83^g^	19.3 ± 0.25^a^	31.4 ± 0.43^bc^	0.0 ± 0.00^c^	0.0 ± 0.00^h^
Olpir	3527 ± 257^cd^	93.4 ± 5.02^d^	248 ± 19.4^cd^	108.6 ± 8.83^d^	56.8 ± 0.99^ef^	15.2 ± 2.97^b^	31.2 ± 4.55^bc^	0.0 ± 0.00^c^	0.0 ± 0.00^h^
Koniz	4452 ± 143^a^	254 ± 20.9^b^	447 ± 0.99^b^	261.4 ± 1.34^b^	119 ± 3.04^b^	18.0 ± 0.68^a^	46.1 ± 1.01^a^	0.41 ± 0.05^b^	16.9 ± 0.95^d^
Rawa	2848 ± 84.1^e^	78.8 ± 4.10^de^	267.4 ± 3.96^c^	105.6 ± 2.97^d^	50.5 ± 1.70^f^	6.26 ± 0.30^c^	24.5 ± 0.16^c^	0.0 ± 0.00^c^	0.0 ± 0.00^h^
Aztek	4250 ± 206^ab^	308 ± 10.3^a^	576 ± 47.0^a^	484.7 ± 17.5^a^	178.4 ± 6.08^a^	14.1 ± 0.50^b^	28.1 ± 0.69^bc^	1.19 ± 0.11^a^	841 ± 17.3^c^
Shapiro–Wilk	0.6328	0.2911	0.2761	0.4994	0.8387	0.00013	0.8114	< 0.00001	0.00098
Bartlett Test	0.1984	0.2871	0.2482	0.5436	0.9081	< 0.00001	0.299	< 0.00001	< 0.00001
Durbin–Watson	0.3562	0.1649	0.2791	0.2583	0.3109	0.3918	0.7357	0.09896	0.2105
Box‐Cox (λ)	—	—	—	—	—	—	—	—	—
Tukey's HSD test	Yes	Yes	Yes	Yes	Yes	No	Yes	No	No
Kruskal–Wallis test	No	No	No	No	No	Yes	No	Yes	Yes

*Note:* Data are expressed as mean ± standard deviation (*n* = 2). *p*‐values are reported for the Shapiro–Wilk (normality), Bartlett (homoscedasticity), and Durbin–Watson (errors' independence) tests. Box–Cox lambda “*λ*” values (−2.00 to +2.00) indicate data transformation for normality or homoscedasticity. Different lowercase letters (^a–g^) denote statistically significant differences among amaranth samples using Tukey's HSD (parametric) or Dunn's test following Kruskal–Wallis (non‐parametric) analysis (*p* < 0.05), depending on assumption acquiescence.

*β*‐sitosterol emerged as the dominant phytosterol, with the highest value found in Aztek (484.7 mg/100 g), while stigmasterol levels ranged from 155 mg/100 g (Kiwicha local market 2) to 576 mg/100 g (Aztek). Both compounds have been associated with cholesterol‐lowering and cardioprotective effects (Das and Kalyani 2024). Nonetheless, factors such as bioavailability, food matrix interactions, and consumer acceptability of amaranth‐based products must also be considered when evaluating the practical value of these lipophilic compounds. Furthermore, a moderate to strong correlation (*r* = 0.657–0.787) between squalene, protein, and certain minerals (Table 5) suggests that targeted cultivar selection and agronomic strategies may enhance both nutritional and functional properties. Still, it is important to recognize that these relationships were derived from a single‐season dataset and may not remain consistent across different environmental conditions or cultivation years. This underlines the need for longitudinal, multi‐location studies to substantiate these findings and guide evidence‐based breeding or utilization strategies.

**TABLE 5:** Linear correlations of proximate analysis (crude protein, starch, fat, ash, dry matter), bioactive compounds (total phenolic acid, total polyphenols, and total flavonoids), DPPH antioxidant capacity, squalene, *α*‐tocopherol, and *β*‐tocopherol among *Amaranthaceae* varieties.

Correlations	Starch	Protein	Flavonoids	Polyphenols	DPPH	Phenolic acid	Dry matter	Ash	Fat	Leucine	Phe	Lysine	Calcium	Magnesium	Iron	Squalene	*α*‐tocopherol
Protein	−0.52																
Flavonoids	0.065	−0.315															
Polyphenols	**0.736** *	−0.586	0.419														
DPPH	−0.125	0.046	0.638	0.405													
Phenolic acid	0.528	−0.243	0.166	0.584	0.473												
Dry matter	0.105	0.569	0.027	0.187	0.382	0.178											
Ash	0.363	0.047	−0.037	0.494	0.189	0.078	0.536										
Fat	**−0.729** *	**0.701** *	−0.025	−0.485	0.322	−0.125	0.204	−0.139									
Leucine	−0.459	**0.858** **	**−0.655** *	−0.626	−0.204	−0.241	0.314	0.076	0.592								
Phe	0.41	0.066	−0.021	0.444	0.42	**0.696** *	0.424	0.579	−0.081	0.159							
Lysine	−0.26	**0.684** *	**−0.843** **	−0.602	−0.494	−0.288	0.171	0.092	0.334	**0.93** ***	0.105						
Calcium	−0.454	**0.644** *	0.14	−0.417	0.196	−0.29	0.439	0.27	0.592	0.42	0.072	0.223					
Magnesium	−0.504	**0.664** *	−0.201	−0.571	−0.186	−0.54	0.223	0.21	0.568	**0.652** *	−0.092	0.538	**0.84** **				
Iron	0.094	0.14	0.647	0.211	0.55	0.368	0.327	−0.188	0.275	−0.265	−0.001	−0.443	0.168	−0.222			
Squalene	−0.062	0.552	**−0.686** *	−0.512	−0.484	−0.253	0.279	0.308	0.146	**0.707** *	0.189	**0.806** **	0.506	**0.657** *	−0.41		
*α*‐tocopherol	0.005	0.647	−0.422	−0.383	−0.207	0.007	0.481	0.321	0.303	0.608	0.322	0.623	**0.653** *	0.578	0.031	**0.871** **	
*β*‐tocopherol	0.553	**−0.654** *	−0.184	0.505	−0.147	0.371	−0.349	0.205	−0.661	−0.273	0.437	−0.09	**−0.681** *	−0.492	−0.54	−0.152	−0.394

*Note:* Values in bold indicate very strong (*p*‐value < 0.001), strong (*p*‐value < 0.01) or moderate (*p*‐value < 0.05) correlation between dependent variables.

* *p* < 0.05.

** *p* < 0.01.

*** *p* < 0.001.

### 3.5 Tocopherols Analysis

Tocopherols (*α‐*, *β‐*, *γ‐*, and *δ*‐) are well‐recognized for their antioxidant activity and vitamin E functionality, with the amaranth samples evaluated here exhibiting diverse and cultivar‐specific profiles (Table 4). For example, *α*‐tocopherol content was relatively high in Slovak cultivars such as Pribina (19.3 mg/100 g) and Koniz (18.0 mg/100 g), while several Peruvian kiwicha samples contained low *α*‐tocopherol but moderately elevated *β*‐tocopherol levels (31–49 mg/100 g). Notably, *δ*‐tocopherol was exceptionally high (1786 mg/100 g) in Kiwicha red and white. These findings are consistent with the notion that distinct metabolic pathways influence the distribution of tocopherol isomers among genotypes, as supported by the strong positive correlation observed between squalene and *α*‐tocopherol (*r* = 0.871), and the inverse correlation between *β*‐tocopherol and protein content (*r* = −0.654) (Zaaboul and Liu 2022).

Tocopherol isomers act synergistically to promote human health through antioxidant and anti‐inflammatory mechanisms. *α*‐tocopherol, the most biologically active form in humans, supports antioxidant defense, immune function, and skin health. *β*‐ and *δ*‐tocopherols also contribute antioxidant effects that may benefit cardiovascular health and cellular protection, while *γ*‐tocopherol is particularly effective at neutralizing reactive nitrogen species and may reduce inflammation and cancer risk (Batool et al. 2020; Szewczyk et al. 2025). However, it is important to note that the bioavailability and metabolic fate of these isomers can differ substantially, and their actual impact depends on dietary context and individual physiology, aspects not addressed in this study. From a nutritional and functional food perspective, tocopherol‐rich amaranth varieties offer promising natural antioxidant sources, suggesting potential for oil extraction or fractionation to develop fortified ingredients (Schnetzler 2018). Nonetheless, challenges remain regarding the stability of tocopherols during processing, variability across harvest seasons, and the need for in vivo validation of their health effects. Therefore, while the data support the food and nutraceutical potential of amaranth tocopherols, further research is warranted to clarify their functional relevance and optimize cultivar selection and processing conditions.

### 3.6 Correlation Analysis and Principal Component Analysis

#### 3.6.1 Correlation Analysis

Starch showed a moderate positive correlation with total polyphenols (*r* = 0.736) but an inverse association with fat (*r* = −0.729) (Table 5), suggesting potential metabolic trade‐offs in carbohydrate and lipid pathways (Baraniak and Kania‐Dobrowolska 2022). Moreover, the positive starch‐polyphenol correlation (*r* = 0.736) may reflect shared sugar precursors (e.g., glucose‐6‐phosphate) that feed both starch biosynthesis and the phenylpropanoid pathway, as demonstrated in amaranth under elevated carbon sink strength (Gómez‐Caravaca et al. 2012). While the inverse correlation between starch and fat aligns with known carbon partitioning in seeds, where genotypes favoring carbohydrate storage often show reduced lipid biosynthesis (Baraniak and Kania‐Dobrowolska 2022). Crude protein positively correlated with leucine (*r* = 0.858) and lysine (*r* = 0.684), confirming that higher‐protein cultivars also feature elevated essential amino acids (Aswal et al. 2025). Squalene displayed moderate to high correlations with *α*‐tocopherol (*r* = 0.871) and lysine (*r* = 0.806), supporting the view that certain amaranth genotypes simultaneously accumulate beneficial lipids and amino acids (Singhania et al. 2023).

#### 3.6.2 Principal Component Analysis (PCA)

**FIGURE 2:**
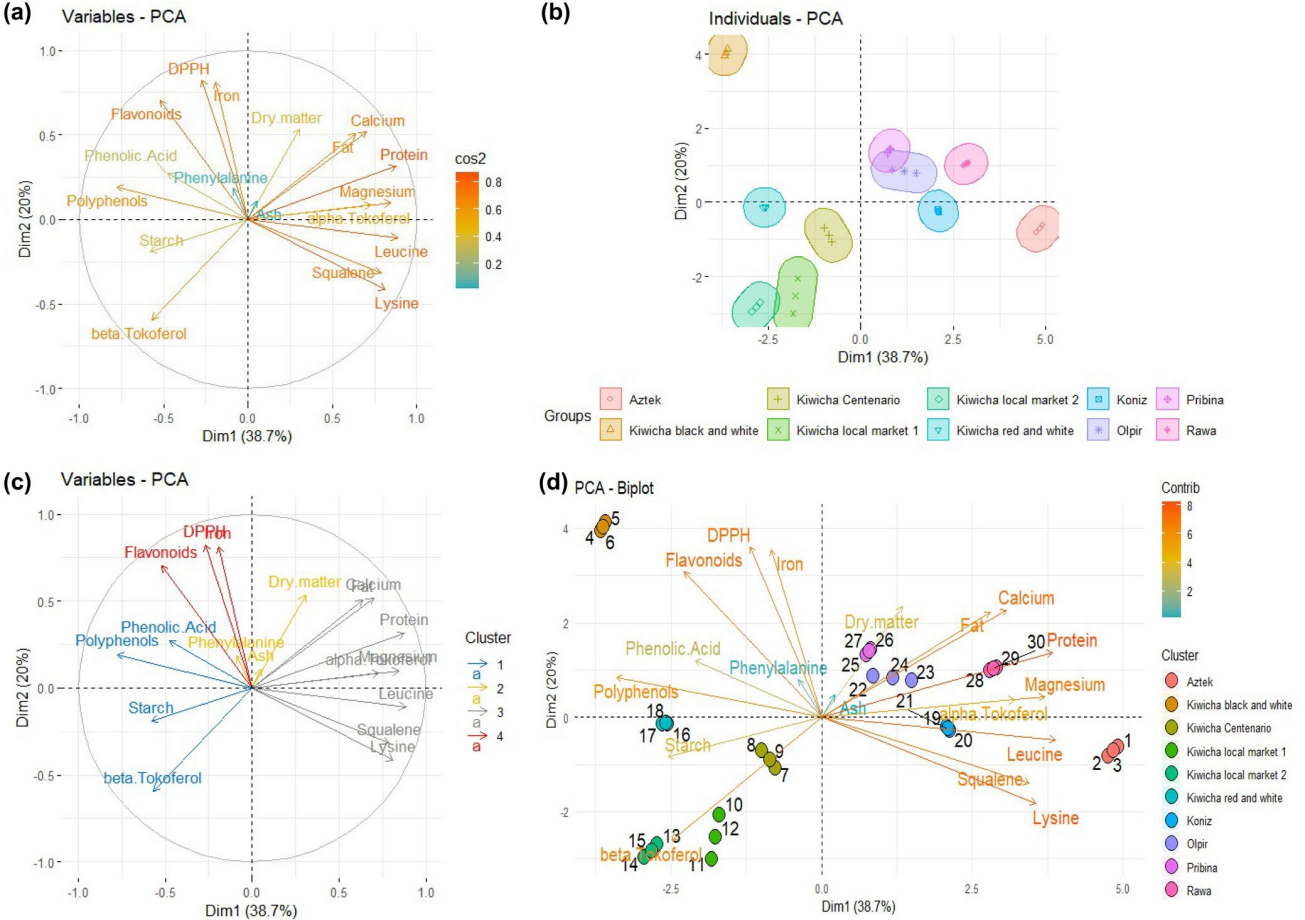
Principal component analysis (PCA) showing two‐factor maps (a, c) and scores projections (b, d) by *Amaranthaceae* varieties (kiwicha black and white, kiwicha red and white, kiwicha local market 1, kiwicha local market 2, kiwicha Centenario, Pribina, Olpir, Koniz, Rawa, and Aztek) proximate analysis (crude protein, starch, fat, ash, dry matter), bioactive compounds (total phenolic acid, total polyphenols, and total flavonoids), DPPH antioxidant capacity, squalene *α*‐tocopherol and *β*‐tocopherol. (a) Variable's graph showing the principal component 1 (PC 1, Dim1 = 38.7%) and PC 2 (Dim2 = 20.0%) variability impact (cos^2^), (b) Individual's graph showing the two PCA (PC 1 and PC 2) for each *Amaranthaceae* variety linking, (c) Variable's graph showing the PC 1 (Dim1 = 38.7%) and PC 2 (Dim2 = 20.0%) clustering, (d) Biplot graph showing the PCA by *Amaranthaceae* varieties and their degree of contribution.

PCA was performed on the combined data set (proximate composition, amino acids, minerals, bioactive compounds, antioxidant activity, and selected lipophilic constituents). The first two principal components (PC1 and PC2) together accounted for 58.7% of the total variance (PC1: 38.7%; PC2: 20.0%), demonstrating their importance in differentiating the amaranth samples (Table 6). As shown in Figure 2a, variables such as crude protein, leucine, and magnesium exhibited high cos^2^ values on PC1, indicating that cultivars with higher protein often also displayed elevated amino acid and mineral levels. Conversely, DPPH radical‐scavenging capacity and iron were principal contributors to PC2, reflecting a secondary axis driven by antioxidant and mineral traits (Zhu 2023).

**TABLE 6:** PC pattern for the first (PC 1), second (PC 2) and third (PC 3) components (dimension), eigen values, variance explained among proximate analysis (crude protein, starch, fat, ash, dry matter), bioactive compounds (total phenolic acid, total polyphenols, and total flavonoids), DPPH antioxidant capacity, squalene, *α*‐tocopherol, and *β*‐tocopherol for *Amaranthaceae* varieties.

Features	PC 1	PC 2	PC 3
Leucine	0.8867	−0.1091	0.1892
Phenylalanine	−0.0872	0.1825	0.8715
Lysine	0.8131	−0.4167	0.2438
Starch	−0.5818	−0.1904	0.6142
Protein	0.8762	0.3148	0.0835
Flavonoids	−0.5207	0.7036	−0.2425
Polyphenols	−0.7784	0.1927	0.4888
DPPH	−0.2717	0.8249	0.1311
Phenolic acid	−0.4784	0.2740	0.5433
Dry matter	0.3069	0.5345	0.5695
Ash	0.0512	0.1094	0.7602
Fat	0.6391	0.5068	−0.2442
Calcium	0.7007	0.5195	0.0522
Magnesium	0.8460	0.1001	−0.0244
Iron	−0.1925	0.8105	−0.1056
Squalene	0.7876	−0.3179	0.4137
*α‐*tocopherol	0.7307	0.0854	0.4841
*β‐*tocopherol	−0.5694	−0.5940	0.4031
Eigen values	6.97	3.59	3.39
Variance, %	38.7	20.0	18.9

Figure 2b illustrates that Aztek and Koniz cluster on the positive side of PC1, exhibiting high protein, leucine, and squalene (Karamać et al. 2019), while Kiwicha black and white stand apart due to moderate protein but comparatively high antioxidant activity. Figure 2c shows protein, leucine, lysine, and squalene forming a distinct cluster, whereas total phenolic acid, total polyphenols, starch, and *β*‐tocopherol group separately. Finally, the biplot (Figure 2d) reveals that Pribina, Olpir, and Rawa align with higher protein and fat contents, whereas Kiwicha Centenario and Kiwicha red and white associate more strongly with *β*‐tocopherol and polyphenol vectors. These patterns demonstrate diverse cultivar profiles and reveal opportunities for selective breeding tailored to specific nutritional or functional goals.

While PCA revealed strong clustering by cultivar (Figure 2a), the cumulative variance (58.7%) indicates additional factors may influence nutritional profiles. For instance, Peruvian accessions, enriched in Fe and antioxidants, formed a separate cluster in PC2, potentially reflecting adaptive responses to high‐altitude stressors. In this context, (Sarker and Oba 2018) demonstrated that drought stress significantly alters amaranth's metabolite allocation, favoring stress‐responsive compounds (e.g., phenolics) over storage proteins or carbohydrates. This underscores the need for future studies integrating field conditions (e.g., UV radiation, micronutrients in soils, growing temperatures, etc.) with compositional analyses to disentangle genetics from environmental effects.

## 4 Conclusion

This study highlights the considerable variability in macronutrients, minerals, amino acids, and bioactive compounds among *Amaranthus* species cultivated in Peru, Slovakia, and Poland, reinforcing its value as a resilient pseudocereal with promising nutraceutical and functional food potential. Polish cultivars demonstrated higher protein and essential amino acid content, while Peruvian kiwicha varieties exhibited elevated levels of polyphenols and antioxidant activity. Mineral composition—including calcium, magnesium, iron, and zinc—varied across cultivars, suggesting both environmental and genetic influences. Notably, Slovak and Polish samples were rich in lipid‐associated compounds such as squalene, phytosterols, and tocopherol isomers, which are relevant for cardiovascular and metabolic health. These findings support the potential of Amaranthus as an ingredient in gluten‐free and nutrient‐enriched food products and point to its relevance in addressing protein and micronutrient deficiencies in vulnerable populations. Notably, the absence of multiseasonal sampling and bioavailability assessments limits the interpretation of the nutritional and bioactive profiles. This highlights the need for further research to comprehensively understand the factors influencing variabilities in amaranth. Future research should focus on seasonal validation, genotype × environment interactions, bioavailability studies, and in vivo investigations to better establish the health effects of amaranth consumption. Moreover, future studies should be conducted to validate detection thresholds and/or possibly use more sensitive methods to confirm these findings for tocopherol isomers. In parallel, the development of novel food formulations can further position amaranth as a strategic crop in global food and nutrition security efforts.

## Author Contributions

**Emmanuel Duah Osei:** conceptualization (equal), formal analysis (equal), investigation (equal), visualization (equal), writing – original draft (equal). **Alfred Elikem Kwami Afedzi:** conceptualization (equal), formal analysis (equal), investigation (equal), visualization (equal), writing – original draft (equal). **Anthony Amotoe‐Bondzie:** visualization (equal), writing – review and editing (equal). **Eva Ivanišová:** conceptualization (equal), data curation (equal), formal analysis (equal), funding acquisition (equal), investigation (equal), methodology (equal), project administration (equal), resources (equal), supervision (equal), validation (equal), writing – review and editing (equal). **Christian R. Encina‐Zelada:** conceptualization (equal), data curation (equal), funding acquisition (equal), project administration (equal), resources (equal), software (equal), supervision (equal), validation (equal), visualization (equal), writing – review and editing (equal). **Sylwester Czaplicki:** methodology (equal), writing – review and editing (equal). **Iveta Čičová:** formal analysis (equal), methodology (equal), project administration (equal), resources (equal). **Ivona Jančo:** formal analysis (equal), methodology (equal), writing – review and editing (equal). **Branislav Gálik:** methodology (equal), validation (equal), writing – review and editing (equal). **Newlove Akowuah Afoakwah:** conceptualization (equal), supervision (equal), validation (equal), writing – review and editing (equal).

## Conflicts of Interest

The authors declare no conflicts of interest.

## Data Availability

Data will be made available by the corresponding author upon request.
